# Effect of HIPEC on Peritoneal Recurrence in Peritoneal Metastasis Treated With Cytoreductive Surgery: A Systematic Review

**DOI:** 10.3389/fonc.2021.795390

**Published:** 2021-12-03

**Authors:** Daniel Ren Yi Yap, Jolene Si Min Wong, Qiu Xuan Tan, Joey Wee-Shan Tan, Claramae Shulyn Chia, Chin-Ann Johnny Ong

**Affiliations:** ^1^ Department of Sarcoma, Peritoneal and Rare Tumors (SPRinT), Division of Surgery and Surgical Oncology, National Cancer Centre Singapore, Singapore, Singapore; ^2^ Department of Sarcoma, Peritoneal and Rare Tumors (SPRinT), Division of Surgery and Surgical Oncology, Singapore General Hospital, Singapore, Singapore; ^3^ Laboratory of Applied Human Genetics, Division of Medical Sciences, National Cancer Centre Singapore, Singapore, Singapore; ^4^ SingHealth Duke-NUS Oncology Academic Clinical Program, Duke-NUS Medical School, Singapore, Singapore; ^5^ SingHealth Duke-NUS Surgery Academic Clinical Program, Duke-NUS Medical School, Singapore, Singapore; ^6^ Institute of Molecular and Cell Biology, A*STAR Research Entities, Singapore, Singapore

**Keywords:** peritoneal metastasis, cytoreductive surgery, hyperthermic intraperitoneal chemotherapy (HIPEC), recurrence, gastric, ovarian, colorectal

## Abstract

**Background:**

Peritoneal metastasis (PM) is a late-stage manifestation of intra-abdominal malignancies. The current standard of care indicates that cure can only be achieved with cytoreductive surgery (CRS) which is often indicated with concurrent adjuvant hyperthermic intraperitoneal chemotherapy (HIPEC). However, the utility of HIPEC within subsets of PM is not fully understood. We seek to compare the effectiveness of HIPEC in improving peritoneal recurrence rates in PM of different origins.

**Methods:**

We conducted a systematic review of trials on the PubMed, EMBASE, and Cochrane databases, last searched in August 2021. Biases were assessed using the Cochrane Collaboration’s tool for assessing the risk of bias in randomized trials as well as the Methodological Index for Non-Randomized Studies (MINORS) framework.

**Results:**

7 gastric PM studies, 3 ovarian PM studies, and 3 colorectal PM studies were included. Recurrence-free survival was improved in the HIPEC + CRS cohort in 5 gastric trials but only 1 ovarian trial and none of colorectal origin.

**Discussion:**

Our findings indicate decent effectiveness of HIPEC in gastric PM, but limited utility in ovarian and colorectal PM. Limitations in the current literature are attributed to the paucity of data available, a lack of homogeneity and consideration of novel and personalised treatment regimens. We implore for further studies to be conducted with a focus on patient selection and stratification, and suggest a reframing of approach towards modern molecular and targeted therapeutic options in future studies of HIPEC.

**Systematic Review Registration:**

https://www.researchregistry.com/browse-the-registry#registryofsystematicreviewsmeta-analyses/registryofsystematicreviewsmeta-analysesdetails/60c1ffff0c1b78001e8efbe3/, identifier reviewregistry1166.

## 1 Introduction

Peritoneal metastasis (PM), often synonymous with the term peritoneal carcinomatosis (PC), often presents as a late-stage manifestation of intra-abdominal malignancies characterized by metastasis of cancer to the peritoneal surface and a resultant dissemination of malignancy in the peritoneal cavity (1). The onset of PM is shown to significantly reduce survival in these patients and its occurrence varies between subtypes, having been approximated from anywhere between 4-18% in colorectal cancer to up to 50% in gastric cancer (1, 2). Historically, peritoneal metastasis was largely considered a lethal disease with little room for curative intent given its lower response rates to normal systemic chemotherapy in comparison to other organ metastases (2). In recent times however, cytoreductive surgery (CRS) with or without hyperthermic intraperitoneal chemotherapy (HIPEC) have seen emergence as an accepted course of treatment, which improves survival and disease burden significantly in PM patients; this renaissance was in particular spearheaded by Dr Paul Sugarbaker (3, 4). Hyperthermic intraperitoneal chemotherapy (HIPEC) is a localized chemotherapeutic treatment recommended as an immediate follow-up to primary cytoreductive surgery for multiple reasons. Of these, its proposed key advantages include superior tissue penetration of the peritoneal cavity alongside an observed anti-tumor cell response given by hyperthermia, eliminating residual microscopic tumors post-cytoreduction in an adjuvant setting, translating to improved survivals (5, 6). In extension, Sugarbaker recently proposed a wide range of PM subtypes whereby CRS and HIPEC utilized together may be recommended, including that of appendiceal cancer, colon cancer, ovarian cancer, and gastric cancer, fueling greater intrigue into its possible influence in treatment options (7). Whilst CRS has since largely been accepted as a mainstay of PM treatment, the use of HIPEC within the various subtypes of PM still remains controversial, given in part by the lack of concrete level I evidence established by well-designed randomized controlled trials. This muddle surrounding the efficacy of adjuvant HIPEC alongside CRS thus represents an area of unmet clinical understanding which we hope to address.

We aim to provide a comprehensive overview of the current literature surrounding the effect of HIPEC alongside CRS in current PM patients and patients with a high risk of PM on peritoneal recurrence within an intent-to-cure setting, with special reference to higher level evidence such as randomized clinical trials (RCTs). While RCTs are recognized as the highest level of clinical evidence available, we recognize biases which might interfere with study results on overall survival; namely, the RCT study selection or inclusion criteria that might bias towards patients of better disease biology or burden. Our primary endpoint focus on peritoneal recurrence is thus intended to serve as a surrogate marker to eliminate additional factors that can confound overall survival results, driven by the hypothesis that decreased peritoneal recurrences will directly contribute to improved overall survival, given that the majority of mortality in PM stems from a relapse of peritoneal metastases.

## 2 Materials and Methods

### 2.1 Search Terms and Data Sources

A literature search was performed for relevant studies, with search terms systematically applied onto the PubMed, EMBASE, and Cochrane databases, respectively. The search was independently conducted by 2 researchers (DRYY, JSMW); where there were discrepancies, a third reviewer finalized the decision (QXT). We settled on four strings of search terms which effectively narrowed our database searches to articles of relevance pertaining to our pre-selected keywords. Search terms 1 and 2 are similar, with term 1 specifying the search in MeSH terms, allowing the search to be more focused; terms 3 and 4 aim to specifically identify if studies with a propensity score-matching (PSM) analysis had been performed on this topic, to obtain high-level cohort studies to supplement our considered list of papers. References in published articles and reviews were screened to identify additional articles where relevant and filters were added to restrict search results to human studies and English-published studies. No time filters were applied to either of the searches and searches were last performed in August 2021. The list of search terms applied are given in **Table 1**.

**Table 1:** Search terms used for literature search.

No.	Search Term
**1**	(Peritoneal Neoplasms[MeSH Terms]) AND (Cytoreduction Surgical Procedures[MeSH Terms]) AND (Hyperthermia, Induced[MeSH Terms]) AND (Neoplasm Recurrence, Local[MeSH Terms])
**2**	((peritoneal carcinomatosis) OR (peritoneal metastasis)) AND ((cytoreductive surgery) AND ((hyperthermic intraperitoneal chemotherapy) OR (pressurized intraperitoneal aerosolized chemotherapy))) AND ((recurrence) or (recurrent))
**3**	(Peritoneal Neoplasms[MeSH Terms]) AND (Cytoreduction Surgical Procedures[MeSH Terms]) AND (Hyperthermia, Induced[MeSH Terms]) AND (Neoplasm Recurrence, Local[MeSH Terms]) AND (Propensity Score[MeSH Terms])
**4**	((peritoneal carcinomatosis) OR (peritoneal metastasis)) AND ((cytoreductive surgery) AND ((hyperthermic intraperitoneal chemotherapy) OR (pressurized intraperitoneal aerosolized chemotherapy))) AND ((recurrence) or (recurrent)) AND (propensity score)

### 2.2 Background Literature Search

Through detailed searches of the relevant databases, we concluded that there are no recent reviews (in either systematic review or meta-analysis form) specifically pertaining to this research question with our specific focus on peritoneal recurrence. Prior studies on HIPEC were predominantly concerned on the effect of HIPEC on overall survival (OS) and often had lacking data to provide a proper analysis of tumor recurrence as a result of treatment (8). Another study from 2013 also touched on tumor recurrence given by HIPEC but was similarly restricted solely to bowel cancer, and only utilized 1-,2-,3-, and 5-year recurrence rates from a small selection of studies which published them (9). Review articles to date thus sparingly touch on this target question or provide limited to no concrete answer. Brenkman et al. (10) also published a recent paper on the effects of HIPEC on gastric cancer with a wider study selection, though the paper expanded their search to consider all comparative studies which thus included case control and cohort studies (10). Similarly, a comprehensive recent systematic review and meta-analysis performed by Cianci et al. conducted on HIPEC in ovarian cancer included case-control studies in its review of the current literature, but this review did yield optimistic results for HIPEC therapy despite noting significant heterogeneity of the evidence studied as a major limitation (11). Two propensity score-based cohort studies, one on ovarian cancer (12) and one on gastric cancer (13), were also reviewed (12, 13). The study on ovarian cancer had data on peritoneal recurrence, but no difference in recurrence rates were observed (p=0.454), while the study on gastric cancer had a significant decrease in peritoneal recurrence in the HIPEC group given by a longer recurrence-free survival (p=0.001).

### 2.3 Study Selection and Synthesis

Only studies with a controlled trial study design were considered, although RCTs were prioritized for evidence during our review of the articles. As an exception, we also conducted a separate search for propensity score-matched cohort studies to supplement our dataset of articles, although recognizing that they consist of a lower level of evidence than clinical trials. Review articles such as systematic reviews and meta-analyses were also considered, their findings and references reviewed to identify additional relevant articles for inclusion. Studies that were not predominantly focused on the effect of additional HIPEC therapy on tumor recurrence or which focused predominantly on other aspects of therapy (such as postoperative chemotherapy in addition to CRS + HIPEC, or which compared HIPEC to a control standard other than ‘gold-standard’ of CRS alone) were promptly excluded. Papers that referenced the same clinical study were removed from our consideration to prevent duplication of study groups. Articles were then manually reviewed by members of the team *via* an assessment of the study characteristics, presence of sub-grouping (with particular reference to peritoneal cancer index (PCI) and completeness of cytoreduction (CC) scores), and trial outcomes including DFS, OS, and peritoneal recurrence rates.

### 2.4 Study Quality Assessment

The quality of the selected RCTs were assessed with reference to the Cochrane Collaboration’s tool for assessing the risk of bias in randomized trials (14). The majority of articles graded well under these guidelines, although most were assessed to have a middle/uncertain or high levels of bias concerning participant-sided blinding due to the nature of the intervention performed on patients. Similarly, the non-randomized controlled trials and propensity score-matched cohort studies were evaluated with help of the Methodological Index for Non-Randomized Studies (MINORS) framework (15). The full evaluation matrix is appended under **Supplementary Tables S1, S2**.

To further ensure the homogeneity of the data from different sources for eventual cross-referencing, we verified that the definitions of certain key terms in each paper were kept to an identical level of consistency. Certain key terms are listed below and further elaborated upon:

#### 2.4.1 ‘Completeness of Cytoreduction Score’

To ensure that tumor recurrence despite HIPEC therapy occurred independent of confounding factors such as an incomplete cytoreductive surgery, all papers considered were screened in an effort to restrict our investigation to a patient base with optimal or near-optimal ‘complete’ cytoreduction (a post-CRS CC score of 0-1). For papers which included a CC scoring system, CC scores were given by the definition laid out by Sugarbaker (16). The paper by van Driel (17) used an alternative R scoring system instead, but the classifications were largely comparable with the ‘R-0’ classification analogous to a ‘CC 0’ parameter laid out by Sugarbaker describing a complete cytoreduction (18).

#### 2.4.2 ‘HIPEC/Hyperthermic Intraperitoneal Chemotherapy’ and Related Variants

Before proper standardization of current HIPEC treatment guidelines, older studies used a set of terms to describe therapy given which are largely similar to the current HIPEC treatment. We thus investigated the procedure methods to ensure that the procedures reflected in these clinical trials were comparable to that of modern HIPEC. Intraperitoneal hyperthermic chemoperfusion (IHCP) as coined by the Fujimoto et al. (19) listed a methodology similar to that of modern HIPEC, with a chemotherapy mixture of mitomycin C perfused at a temperature of 44.5-45.0°C (19). Continuous hyperthermic peritoneal perfusion (CHPP) as indicated by Koga et al. and Fujimura et al. were also described as an intraperitoneal hyperthermic perfusion of mitomycin C at 44-45°C and 41-42°C respectively (20, 21). We are aware of the immense variability in methodology between various HIPEC treatment options, given by prior established reviews into the heterogeneity of HIPEC regimens used in clinical trials (22, 23). Given that the studies converge upon the basis of intraperitoneal chemotherapeutic infusion being performed explicitly as a means of targeting peritoneal metastases, these terms were thus assessed to be analogues of ‘HIPEC’ and were thus treated interchangeably.

#### 2.4.3 ‘Open’ and ‘Closed’ HIPEC Techniques and Methodology

The procedure for HIPEC is performed upon adequate conclusion of tumour cytoreduction and has historically been performed *via* various methodologies which fall within two broad categories: an open abdomen technique and a closed abdomen technique (24). The open, laparotomic, or ‘Coliseum’ technique is often performed as described by Sugarbaker in 2005 whereby the patient’s abdominal skin edges are suspended by a retractor apparatus alongside the integration of a silicon sheet to establish an open space for perfusion of the hyperthermic chemotherapy solution (25). The closed or laparoscopic technique instead involves closure of the abdominal wall after completion of open CRS or is performed after previous laparoscopic CRS with subsequent infusion of the hyperthermic chemotherapy solution into the sealed abdominal compartment (24).

The hypothesised advantages and disadvantages of these techniques have since been well discussed within the academic community. The open technique has been mentioned to give rise to a better distribution of the hyperthermic chemotherapy solution as well as heat across the peritoneal compartment but presents with the downside of intractable heat loss from the chemotherapeutic solution during the procedure, in itself a huge problem due to the inherent narrow therapeutic index of the hyperthermic effect (26). On the other hand, the closed technique provides superior prevention of heat loss and drug spillage with a secondary theorised upside of increased drug penetration, despite the inefficient distribution of perfusion fluid in this method (27). A ‘semi-open’ method with help of a device coined the Peritoneal Cavity Expander (PCE) has also been described by Fujimura with the aim of bridging the benefits from both methods, to minimise the loss of heat while maintaining homogeneity of infusion (28). This, however, does present as a complex setup which is heavily dependent on operator expertise (24).

Despite these hypothesised differences being discussed within academic circles for over a decade, concrete statistical data to denote significant differences in outcomes between the various techniques have remained sparse. Whilst a study by Elias et al. (29) mentioned that they found it impossible to obtain thermal homogeneity in closed laparoscopic HIPEC therapy, a more recent study by Silva et al. (30) found that patient systolic and diastolic pressures were more stable using a closed technique, alongside an association with a reduced number of post-operative complications as well as significantly favourable abdominal temperature. It is thus apparent that there is no current consensus within literature on the superiority of either technique option for HIPEC therapy – the newer phase III trials such as those considered in this publication (COLOPEC, PROPHYLOCHIP, PRODIGE 7) have also since clustered both closed and open HIPEC therapeutic standards into a singular group for discussion. We have thus assessed these two groups to be adequately homologous equivalents of each other based on the literature available at the given moment, which will likely remain the de-facto assumption at least until the advent of future higher powered randomised controlled trials to aid in our understanding of this topic.

**Figure 1:**
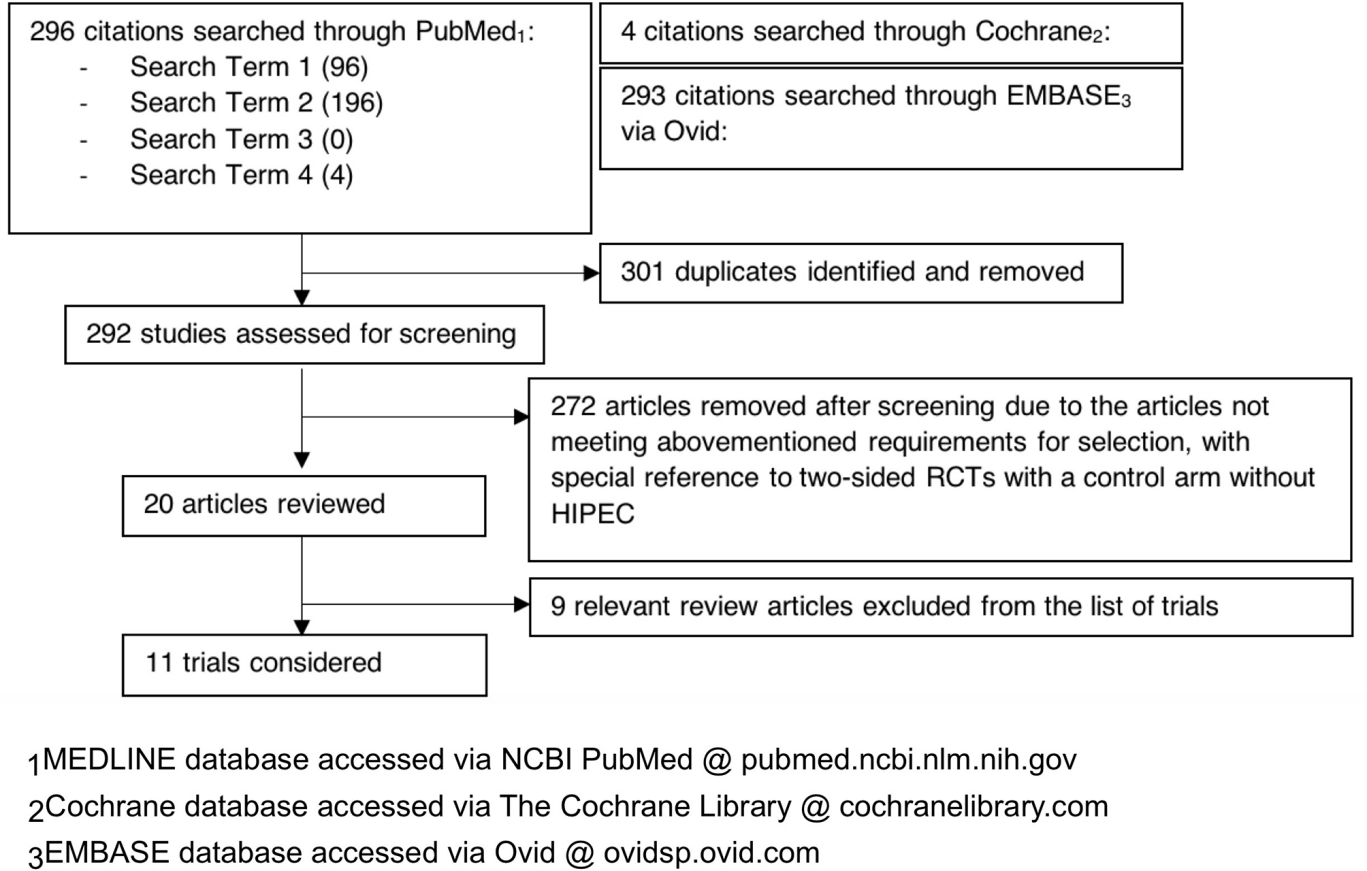
PRISMA flowchart denoting our literature search strategy.

Our literature search flow has been summarized by a PRISMA flow chart in **Figure 1** for reference. This systematic review study is registered with the Research Registry (unique identifying number: reviewregistry1166, https://www.researchregistry.com/browse-the-registry#registryofsystematicreviewsmeta-analyses/registryofsystematicreviewsmeta-analysesdetails/60c1ffff0c1b78001e8efbe3/) (31).

## 3 Results

### 3.1 Gastric Cancer

Five RCTs and one non-randomized CT were reviewed for gastric cancer, though these studies considered were largely older papers. Five studies were predominantly based on a chemotherapeutic regimen of mitomycin C, although the singular non-randomized clinical trial used a unique mixture of mitomycin C, cisplatin, and etoposide. One propensity-matched cohort study was also considered, which instead considered multiple chemotherapy regimens. The papers all reported similarly along the lines that HIPEC treatment reduced the risk of peritoneal recurrence, though some papers struggled to achieve significance for this result, and others extrapolated this conclusion from overall survivals and the percentage of patients which died due to recurrence. Extended median OS or higher 3/5-year survivals were reported for the cohort that underwent HIPEC. Due to the relative age of the papers however, the quality of the data was low; no papers established the extent of tumor resection before inclusion in the trial despite assumed best attempts at cytoreduction of tumor, nor did any mention PCI scores or factors for narrowing of the patient selection criteria. The sole exception was the most recent propensity score-matched study by Bonnot et al. (13), whereby both CC and PCI scores were utilized in propensity score matching and patient selection. Yang et al. however did shed some light on the utility of HIPEC in a relatively more modern setting, indicating significantly improved OS in the HIPEC group, alongside significant improvements in the HIPEC group particularly in patients with lower PCI scores indicating lower PM burden (32). The older RCTs additionally had no information pertaining to disease- or recurrence-free survivals; this lapse paired with the missing significance data of OS and peritoneal recurrence endpoints thus presenting with a significant risk for reporting bias. Despite these omissions, follow-up time of the studies were decent with a median of 30 odd months after surgery, with evidence pointing towards significantly improved OS and peritoneal recurrence rates. The summary of all articles pertaining to gastric cancer is listed in **Table 2**.

**Table 2:** Summary of articles on gastric cancer.

Study	Chemotherapeutic Agent	Primary Investigation	Cohort Characteristics	Study cohort	Impact on OS	Impact on DFS	CC Coverage	PCI Coverage	Main Findings (with focus on peritoneal recurrence)
Koga et al., 1988 (20)	Mitomycin C	**RCT** CRS + HIPEC vs CRS alone (CRSa)	Macroscopic serosal invasion with no macroscopic PM	47	Improved 3- and 5-yr OS in HIPEC group, though no data on significance	Not covered	Not covered	Not covered	CRS + HIPEC had fewer peritoneal recurrences than CRSa (36% v 50%), no data on significance.
Hamazoe et al., 1994 (33)	Mitomycin C	**RCT** CRS + HIPEC vs CRSa	Macroscopic serosal invasion with no macroscopic PM	82	No significance in mean and 5-yr OS in HIPEC group, failed to achieve significance	Not covered	Not covered	Not covered	HIPEC group had fewer peritoneal recurrence, p=0.0854, failed to achieve significance.
Fujimura et al., 1994 (21)	Mitomycin C	**RCT** CRS + HIPEC vs CRSa	Advanced GC and serosal invasion, no mention of PM status	58	Improved 1/2/3-yr OS in HIPEC group, p<0.01	Not covered	Not covered	Not covered	Improved OS and reduced deaths in HIPEC group attributed to reduced peritoneal recurrence. No direct observation of recurrence rates.
Fujimoto et al., 1999 (19)	Mitomycin C	**RCT** CRS + HIPEC vs CRSa	Macroscopic GC without macroscopic PM	141	Improved 2/4/8-yr OS in HIPEC group, p=0.0362	Not covered	Not covered	Not covered	Reduced peritoneal recurrence in HIPEC group, 1.4% vs 23%, p<0.001.
Hirose et al., 1999 (34)	Mitomycin C, cisplatin, and etoposide	**Non-random CT** CRS + HIPEC vs CRSa	Advanced GC with macroscopic serosal invasion, majority with synchronous PM	92	No significance in median and 1-yr OS in HIPEC group, failed to achieve significance (p=0.11)	Not covered	Not covered	Not covered	Reduced peritoneal recurrence in HIPEC group, patients alive without recurrence -> 40% in HIPEC vs 10% in control, p=0.018.
Yang et al., 2011 (32)	Mitomycin C and cisplatin	**RCT** CRS + HIPEC vs CRSa	GC with synchronous PM	68	Improved OS in HIPEC group (p=0.029)	Not covered	Significantly improved OS in HIPEC group for CC0-1 vs CC2-3 (p=0.000)	Significantly improved OS in HIPEC group compared to CRSa group in high PCI subgroup (p=0.012), no significance between groups in low PCI group (p=0.464)	No specific mention of peritoneal recurrence, 79% of patients from the HIPEC group died to progressive intestinal obstruction by PM recurrence or progression, compared to also 79% in the CRSa group.
Bonnot et al., 2019 (13)	No standardized agent for cohort. Monochemotherapy agents include mitomycin C, cisplatin, oxaliplatin, and doxorubicin	**PSM Cohort Study** CRS + HIPEC vs CRSa	GC with limited synchronous PM	277	Improved OS in HIPEC treated group, IPTW-adjusted log-rank p=0.005	Improved RFS in HIPEC group, IPTW-adjusted log-rank p=0.001	Only CC 0-1 considered for selection. Used for PS matching.	Used for propensity-score matching	Improved RFS in HIPEC group, given by an IPTW-adjusted log-rank of p=0.001 and an improved 3- and 5-year recurrence-free survival rates of 5.87% and 3.76% in the HIPEC group vs 20.40% and 17.05% respectively. (p=0.001)

### 3.2 Ovarian Cancer

Two RCTs and a single propensity score-matched study were reviewed pertaining to ovarian cancer. The study by van Driel et al. (17) utilized cisplatin as the chemotherapeutic agent alongside sodium thiosulfate as a means of preventing renal impairment while the studies by Spiliotis et al. (35) and Ceresoli et al. (12) used a combination of both cisplatin and paclitaxel. Overall survival was found to be significantly improved in the HIPEC group; however, it is important to note the heterogeneity of the dataset when interpreting the findings. The study by Spiliotis et al. (35) included a patient cohort presenting with recurrent OC as compared to the two other studies which featured primary OC; the studies by van Driel et al. (17) and Ceresoli et al. (12) additionally utilised neoadjuvant chemotherapy followed by interval debulking surgery as the precursor to HIPEC therapy whereas the study by Spiliotis et al. (35) performed CRS in its standard methodology. Completeness of cytoreduction (CC scores) were considered by Spiliotis et al. (35) and Ceresoli et al. (12) according to Sugarbaker’s specifications; Driel et al. (17) utilized an alternative R scoring system with R-1 indicating no visible tumors after cytoreduction (complete cytoreduction), comparable to CC-0 as defined *via* the CC classification system. Driel et al. (17) identified median recurrence-free survival to be better in the HIPEC group although no data was shared on the significance of this presentation, whilst the propensity score-matched study by Ceresoli et al. (12) on the other hand explicitly failing to identify any significance with regards to the effect of HIPEC on peritoneal recurrence despite similarly identified improvements in overall survivals. PCI scores were considered in the paper by Ceresoli et al. (12), noting that survival in the HIPEC versus the non-HIPEC group was significantly higher, both in PCI<15 (p=0.031) and PCI>15 (p=0.049), though the PCI score was not utilized for consideration of patient selection for HIPEC treatment. Median follow-up times for the studies were optimal with a range between 4.7 – 8.0 years, and results were all reported with adequate coverage of the statistical significance between population endpoints. The summary of the three articles pertaining to ovarian cancer is listed in **Table 3**.

**Table 3:** Summary of articles on ovarian cancer.

Study	Chemotherapeutic Agent	Primary Investigation	Cohort Characteristics	Study cohort	Impact on OS	Impact on DFS	CC Coverage	PCI Coverage	Main Findings (with focus on peritoneal recurrence)
Spiliotis et al., 2014 (35)	Cisplatin and paclitaxel	**RCT** CRS + HIPEC vs CRSa	Recurrent OC with locally advanced disease (FIGO IIIc/IV)	120	Improved median OS in HIPEC group, 26.7 vs 13.4 months, p=0.006	Not covered	CC 0-2 included. OS significantly higher in CC 0, 30.9 vs 16.9 months, p=0.038	Mentioned, but not used as criteria for selection	No specific mention on recurrence
Driel et al., 2018 (OVHIPEC) (17)	Cisplatin	**RCT** CRS + HIPEC vs CRSa	Primary OC with significant abdominal disease/PM	245	Improved median OS in HIPEC group, 45.7 vs 33.9 months. Hazard ratio, p=0.02	Failed to achieve significance, RFS in HIPEC group, 14.2 vs 10.7 months	R-1 considered (complete cytoreduction, equivalent to CC 0)	Not covered	Reduced peritoneal recurrence in the HIPEC group given by increased median recurrence-free in the surgery-plus-HIPEC group (14.2 months vs. 10.7 months).
Ceresoli et al., 2018 (12)	Cisplatin and paclitaxel	**PSM Cohort Study** CRS + HIPEC vs CRSa	Primary OC with locally advanced disease (including PM)	56 after matching	Improved median OS in HIPEC group, no median reached in OS vs median 32.53 in control. (p=0.048)	No significance, median DFS of 13.96 months in HIPEC vs 13.23 months. (p=0.454)	CC 0-3, CC scores used for PS matching. 93% of HIPEC cohort was CC0.	Mentioned, but not used for selection nor PS matching	No significance in peritoneal recurrence amidst significantly improved OS in HIPEC. Peritoneal recurrence of 75% in HIPEC vs 82.1% in control (p=0.515), median DFS of 13.96 in HIPEC vs 13.23 months (p=0.454).

### 3.3 Colorectal Cancer

Three RCTs were reviewed pertaining to colorectal cancer, consisting of the large-scale COLOPEC (36), PROPHYLOCHIP (37), and PRODIGE 7 (38) studies. The findings of these studies have since sparked debate, with results indicating a stark ineffectiveness of oxaliplatin-based HIPEC regimens in colorectal cancer. The median follow-up period was 63.8 months in the PRODIGE 7 study and 50.8 months in the PROPHYLOCHIP study but was not mentioned in the COLOPEC study, and the PRODIGE 7 study was limited to patients with a PCI score of ≤25 (median PCI 10). The PRODIGE 7 and COLOPEC studies utilized oxaliplatin as the chemotherapeutic agent for HIPEC in conjunction with adjuvant systemic chemotherapy administered to both groups of the RCT, whereas the PROPHYLOCHIP study administered varying regimens of HIPEC according to individual patient’s status and tolerance, with patients with highest neurotoxicity levels being treated with mitomycin HIPEC monotherapy instead contrasted to a control group subjected only to regular surveillance. The COLOPEC study established its primary endpoint as peritoneal disease-free survival at 18 months; this parameter failed to achieve significance between the two groups (18-month peritoneal-free survival in HIPEC group 80.9% vs 76.2% in control, p=0.280). Similarly, the PROPHYLOCHIP study failed to achieve significance in all parameters with no significance in recurrence-free, disease-free, and overall survival rates at both 3- and 5-year points. Additionally, the PRODIGE 7 study yielded surprising findings against HIPEC, identifying an increase in complications resulting in elevated grade 3-5 morbidity in the HIPEC group (p=0.035) after 60 days of treatment, with no significant benefit established in median OS, recurrence-free survivals (RFS), nor peritoneal-free survivals. Despite this, *post-hoc* subgroup analysis showed increased median overall and relapse-free survival benefits in the HIPEC + CRS groups in patients with a PCI score of 11-15. The results of these studies were reported with sufficient coverage of the statistical significance between populations. Given the context of the reported trials, the use of HIPEC with oxaliplatin in colorectal cancer did not appear to confer clinical benefits to patients. The results are summarized in **Table 4**.

**Table 4:** Summary of articles on colorectal cancer.

Study	Chemotherapeutic Agent	Primary Investigation	Cohort Characteristics	Study cohort	Impact on OS	Impact on DFS	CC Coverage	PCI Coverage	Main Findings (with focus on peritoneal recurrence)
Klaver et al., 2019 (COLOPEC) (36)	Intraperitoneal oxaliplatin with adjuvant systemic fluorouracil and leucovorin	**RCT** CRS + systemic chemo + HIPEC vs CRS + systemic chemo	Advanced (T4N0-2M0) primary CC without PM	204	No significance in 18-month OS (93.0% in HIPEC vs 94.1% in control), p=0.82	No significance in 18-month DFS (69.0% in HIPEC vs 69.3% in control), p=0.99	Not covered	Not covered	No difference in peritoneal-free survival at 18-months (80.9% HIPEC vs 76.2% control), one-sided log-rank p=0.28.
Goere et al., 2020 (PROPHYLOCHIP) (37)	Oxaliplatin or oxaliplatin + irinotecan + IV fluorouracil or mitomycin-HIPEC	**RCT** CRS + systematic second-look surgery + HIPEC vs CRSa	Advanced primary CC with synchronous PM or established to be at high risk of subsequent PM	150	No significance in 3-year/5-year OS (79%/68% in HIPEC vs 80%/72% in control).	No significance in 3-year/5-year DFS (44%/42% in HIPEC vs 53%/49% in control), p=0.82/0.82	Not covered, 4 patients had advanced peritoneal disease not amenable to resection on second-look surgery	Not covered	No difference in 3-year peritoneal recurrence-free survival (59% HIPEC vs 61% CRSa),
Quenet et al., 2021 (PRODIGE 7) (38)	Oxaliplatin	**RCT** CRS + systemic chemo + HIPEC vs CRS + systemic chemo	Advanced primary CC with synchronous PM	265	No significance in median OS (41.7 in HIPEC vs 41.2 months in CRSa)	No significance in median RFS at 1 year (13.1 months in HIPEC vs 11.1 months in control), p=0.43	Completeness of cytoreduction was considered during randomization	Not randomized by PCI, though PCI ≤ 25 criteria was used for selection	No significant difference in peritoneal-free survival at 48 months (HR 0.908, 95CI 0.74-1.12), long-rank p=0.370. Median overall and relapse-free survival longer in subgroup of patients with PCI 11-15.

### 3.4 Assessment of Study Heterogeneity, Sensitivity Analysis, and Certainty of Evidence

Clinical heterogeneity was apparent within the diverse spread of trials which were considered. The chemotherapeutic regime used in each trial varied according to local treatment guidelines and preferences of each research team, thus increasing the heterogeneity within each comparison study and in extension, reflecting a conclusion which is less direct and collaborative in informing us of the specific utility of a particular formulation of HIPEC the given context. Endpoints considered by the various trials were also varied significantly between researchers, which we mitigated *via* consideration and comparison of specifically defined endpoints instead (peritoneal recurrence rates, OS, DFS).

Given our decision to include PSM cohort studies as a method of supplementing our sources, we additionally performed a sensitivity analysis by considering the subset of data which only included RCTs, effectively excluding the PSM cohort studies from our analysis. As this change only concerned the analysis of gastric and ovarian PM, we observed that the general rhetoric was not changed by the exclusion of these studies. Considering the RCTs independently, OS and peritoneal recurrence rates were still favored in the HIPEC group within both gastric and ovarian subgroups. The removal of the PSM study on gastric cancer by Bonnot et al. (13) however did exclude the only data source with information on recurrence-free survival in gastric cancer, and thus this statistic should be interpreted with discretion.

Finally, we assessed our preliminary spread of evidence with reference to the Grading of Recommendations, Assessment, Development and Evaluations (GRADE) guidelines for evaluation of certainty (39). The studies on ovarian and colorectal cancer were assessed to provide evidence of adequate confidence although the evidence pertaining to gastric cancer was of lower certainty, largely due to limitations including the utilization of older methodology, the failure to consider patient disease characteristics such as CC and PCI scores alongside issues impeding precision and the possibility of inherent reporting bias. Certain pertinent points such as methodological limitations of selected trials which are discussed below in the discussion were also factored into our evaluation. While certain aspects of the GRADE criteria reiterate points made earlier in our analysis of results, the full assessment of the certainty of evidence can be reviewed under **Supplementary Table S3**.

## 4 Discussion

Our literature review covered 13 high level trials, of which 10 were RCTs, one non-randomized controlled trial, and two propensity score-matched cohort studies. These reviews were focused on three subtypes of peritoneal metastases, arising from gastric, ovarian, and colorectal cancer. The studies on gastric and ovarian cancer indicated a level of effectiveness of HIPEC in improving both recurrence rates and overall survivals; in contrast, the studies on colorectal cancer returned low significance on both the effect of HIPEC on recurrence rates and overall survivals.

At a quick glance, a key concern raised from our review is the apparent lack of homogeneity between the clinical trials. Though our review of clinical trials concerning gastric cancer lent general support towards favoring the use of HIPEC therapy, the findings were occasionally inconclusive, suggesting a correlation in the right direction whilst marginally falling short of achieving statistical significance at a level of p<0.05. Furthermore, data pertaining to the statistical power of these trials and the quality of associated data were important pointers which were largely unavailable in the trials concerning gastric cancer. The multitude of RCTs touching on the effectiveness of HIPEC for gastric cancer were older, 20-years old papers published in East Asia (Japan) and thus a poorer representation of HIPEC given the influx of recent evidence guiding current-day protocol in the administration of modern HIPEC. Possibly due to the age of the trials considered, these papers also fail to consider critical parameters which lend weight to therapy outcome or patient selection such as the patients’ CC and PCI scores, with the average study conducting patient selection solely *via* arbitrary means. Similarly, mitomycin C was the choice chemotherapeutic agent utilized in the majority of GC studies – it would be intriguing to see the effect of other chemotherapeutic agents on GC patients, of which cisplatin has shown considerable promise in PM of ovarian origin and in more recent trials concerning GC. As previously mentioned, the implications of substantial heterogeneity within our data include a poor quality of evidence; despite this, the current available evidence points towards an effectiveness in mitomycin C-based HIPEC for the treatment of PM with gastric origin, though the data on patient selection and suitability for HIPEC calls for further scrutiny. An additional intriguing point for consideration stems from Yang et al. (32), which illustrated a key significance in OS within the HIPEC group, particularly concerning the subgroup of patients with reduced peritoneal burden (PCI ≤ 20, suggesting promise for HIPEC in selected subgroups of patients with features which predict sensitivity.

The studies surrounding ovarian cancer had more comprehensive coverage of patient characteristics due to their relative recency, including CC and PCI scores and the extent to which these were used in the various study analyses. These studies were largely based on a chemotherapeutic regimen of cisplatin, with or without paclitaxel. All three studies displayed significant improvement in overall survivals with HIPEC usage, though this result came with a slight puzzle: despite this improvement, none of the studies managed to achieve statistical significance with respect to disease-/recurrence-free survivals or peritoneal recurrence rates. CC scoring and PCI scoring, where covered, were not considered in patient selection nor randomization, which might play a role in accentuating the disparity between peritoneal recurrence rates and eventual overall survival. The studies considered were also not entirely homogeneous in terms of study population characteristics: the van Driel and Ceresoli studies predominantly concerned patients with primary OC with advanced disease including that of PM, whereas the study by Spiliotis et al. included patients with locally advanced recurrent OC with no pre-existing PM. We also acknowledge the documented methodological limitations pertaining to the Spiliotis et al. study, of which include its failure to comply with CONSORT standards and its lack of adequate statistical analysis documentation to prove adequate power of the given trial, which provides an illustration of the current climate reflecting a lack of high-level quality trials instrumental to a comprehensive analysis of treatment outcomes (40). Despite these pointers, we believe that cisplatin-based HIPEC still seems promising in the setting of ovarian-based PM, of which recent studies have indicated promising efficacy and safety profiles of cisplatin as well as oxaliplatin HIPEC in subgroups of OC (41, 42).

The final three RCTs surrounding PM in colorectal cancer (CRC) have generated much debate given the unexpected nature of the presented findings. While the PRODIGE 7 trial investigated a patient cohort with existing PM with a predominant focus on PM treatment and the COLOPEC and PROPHYLOCHIP studies focusing on the prevention of PM development and recurrence, all three trials eventually revealed unfavorable results towards therapy involving HIPEC. Not only did the trials fail to identify any improvements in OS, DFS or peritoneal recurrence under HIPEC therapy consisting of oxaliplatin, the PRODIGE 7 study went further to establish a finding of increased early-stage morbidity in the HIPEC test group. In terms of study quality, the PRODIGE 7 study also stood out with a comprehensive coverage of CC scores which it utilized for randomization purposes, as well as enacting a PCI ≤ 25 criteria for patient selection to eliminate the possibility of result bias from patients with overly pessimistic prognostic factors. Despite what appears to be overwhelming evidence against HIPEC in colorectal cancer however, there remains room for further scrutiny of the data. Firstly, all trials were conducted with oxaliplatin as the primary chemotherapeutic agent; critically, the science behind the specific HIPEC regimen that was investigated by the PRODIGE 7 study has been criticised to be flawed as a choice of chemotherapeutic regimen in the given target population (43). While oxaliplatin is widely defined as a gold standard for CRC given under IV chemotherapy, it is important to consider the efficacy of other cocktail regimens of agents in this distinct context of HIPEC. Secondly, while HIPEC might be assessed to be disadvantageous when applied across a general population, it remains to be seen if personalized treatment would be better received in a curated group of patients. From a clinical standpoint, the PRODIGE 7 study has presently given us a glimpse into the utility of HIPEC in specific subsets of patients with average peritoneal disease burdens (PCI 11-15) on a backdrop of favorable CC scores, indicating promise for a more detailed use case for PCI scores beyond its singular role as an exclusion criterion for overcomplicated PM; this specific utility also being corroborated by the Yang et al. study pertaining to GC. While there is no current gold standard involving molecular markers as a predictor of HIPEC success, ongoing research has also indicated promise in predicting the efficacy of mitomycin-C-based CRS-HIPEC therapy *via* biomarkers; a precise patient selection process given by molecular marker analysis combined with a novel trial of MMC-based HIPEC in colorectal PM could be a promising direction for subsequent high level clinical trials (44, 45). Certain papers have already begun progressing in this direction, elucidating clinical and histopathological factors which might predict the success of HIPEC (46). One study went further to develop a prognostic model termed the colorectal peritoneal metastases prognostic surgical score (COMPASS), which has shown favourable accuracy in its predictions as compared to the previous Peritoneal Surface Disease Severity Score (PSDSS) which it improved upon (47). These prognostic nomograms assist in clinical decision making and seek to direct clinical judgements towards a framework of individualised treatment regimens based on patient risk-factor stratification (48). We thus believe that whilst the current evidence does appear dismal, it would be too hasty to conclude HIPEC as ineffective at this point of time, and reiterate the notion that further high-level studies are required in this area of unmet clinical knowledge with a particular focus on well-defined molecular-based patient selection and stratification guidelines. Promising trials are also currently ongoing, and we look forward to the publication of Phase III trials such as CHIPOR (NCT01376752), CHIPPI (NCT03842982), OVIHIPEC-2 (NCT03772028) for ovarian cancer and CAIRO6 (NCT02758951) for colorectal cancer, which will undoubtedly be useful in expanding our repertoire of evidence regarding this treatment regimen.

The last two to three decades have generated much optimism in the treatment of peritoneal metastasis, with trials surrounding cytoreductive surgery and HIPEC reporting varied degrees of improvements in patient survival and tumor recurrences. This is also supported by prospective nonrandomized studies demonstrating the effectiveness of HIPEC (49, 50). Despite these findings, the medical community to date is still not fully convinced of the effectiveness of HIPEC in conjunction with CRS. Recent advancements in systemic chemotherapy, even in the novel setting of the Pressurized Intraperitoneal Aerosol Chemotherapy (PIPAC) regimen drive hopes for adequate management of this disease but to this date, whilst such regimens have been proven feasible and manageable, the data on its utility and documentation of a recognized, standardized protocol for reproduction is still gravely absent (51).

Crucially, we also observe a distressing paucity of data relating to the usage of adjunct small molecule therapy in the treatment of peritoneal metastasis, especially considering the radical shift in paradigm brought about by the influx of targeted therapy treatment options in the last few decades. We thus need to question if our current standard of treatment for PM is perhaps too primitive of an option for current times. The summary given by our review of current literature is therefore ultimately a call for further research, and an opportunity for consolidation and incitement of a novel direction for future research.

As existing literature has suggested, it is now ever so much more important to consider personalized treatment plans for patients with PM moving forward, which incorporates both clinical and molecular stratification into the patient selection process. As demonstrated in various cancer types, the discovery of prognostic gene signatures could provide clinicians with tools for better disease management (52–54). The potential utility of unique signatures on extracellular vesicles and glycans could also be harnessed for patient prognostication (55). Minimally invasive surgery options in the background of recurrent ovarian cancer treated with HIPEC have recently been suggested as a means of reducing surgical risk and post-operative complications in the subgroup of patients indicated for such surgery, providing desirable outcomes with a reported upside of increased peritoneal uptake of HIPEC (56, 57). Taking it a step further, novel studies pertaining to the tumor cell and tumor environment *via* next generation sequencing and the identification of signaling pathways is required to improve the current approach towards patient therapy, with the goal of outlining possible therapeutic advantages *via* the identification of exploitable biological channels in the pathogenesis of PM. Such a process could open possibilities for unorthodox experimental procedures including the likes of targeted therapy as a replacement for conventional chemotherapy in the setting of hyperthermic intraperitoneal perfusion. Eventually, the end goal is to facilitate a marriage between the current knowledge of conventional clinicopathologic prognostic variables and novel molecular-level treatment pathways to produce the optimal therapeutic course for patients. This will then ultimately require large-scale assessment *via* a systematic multi-center approach such as that of the impressive RENAPE observational registry – a specialised retrospective, longitudinal patient registry which incorporates the details and outcomes of patients with rare peritoneal malignancies within key treatment institutions throughout France to facilitate the study of outcomes in such rare diseases, along with sufficient and diverse studies to follow up on the inquiry into these aspects of therapy and management (58). A strategy in this direction is ideally the most comprehensive method to inspire continued advancement in our understanding of this uncommon condition.

## Data Availability Statement

The original contributions presented in the study are included in the article/**Supplementary Material**. Further inquiries can be directed to the corresponding author.

## Author Contributions

Conceptualization, C-AJO. Methodology, DRYY and JSMW. Validation, DRYY, JSMW, QXT, and JW-ST. Formal analysis, DRYY and JSMW. Investigation, DRYY, JSMW, QXT, and JW-ST. Resources, CSC, and C-AJO. Data curation, DRYY and JSMW. Writing – original draft, DRYY, JSMW, QXT, and JW-ST. Writing – review and editing, DRYY, JSMW, QXT, JW-ST, CSC, and C-AJO. Visualization, DRYY and QXT. Supervision, JSMW, CSC, and C-AJO. Project administration, DRYY, QXT, and JW-ST. Funding acquisition, C-AJO. All authors contributed to the article and approved the submitted version.

## Funding

This study is supported by the NCCS Cancer Fund (Research) and SingHealth Duke-NUS Academic Medicine Centre, facilitated by Joint Office of Academic Medicine (JOAM). As part of the Singapore Gastric Cancer Consortium, the study is also partially funded by the National Medical Research Council Open Fund-Large Collaborative Grant (OFLCG18May-0023). C-AO is supported by the National Research Council Transition Award (NMRC/TA/0061/2017). All the funding sources had no role in the study design, data interpretation or writing of the manuscript.

## Conflict of Interest

The authors declare that the research was conducted in the absence of any commercial or financial relationships that could be construed as a potential conflict of interest.

## Publisher’s Note

All claims expressed in this article are solely those of the authors and do not necessarily represent those of their affiliated organizations, or those of the publisher, the editors and the reviewers. Any product that may be evaluated in this article, or claim that may be made by its manufacturer, is not guaranteed or endorsed by the publisher.
