# Supplementary material for: Effect of HIPEC on Peritoneal Recurrence in Peritoneal Metastasis Treated With Cytoreductive Surgery: A Systematic Review
Source: Front Oncol. 2021 Dec 3;11:795390. doi: 10.3389/fonc.2021.795390 (PMC8678115; doi:10.3389/fonc.2021.795390)
Supplement: Supplementary file 1 [file Table_1.docx]

Supplementary Material

# Supplementary Table

**Supplementary Table S1** – Summary of quality assessment rubrics given by the Cochrane Collaboration’s tool of assessing risk of bias in randomised trials

| No. | Assessment Question |
| --- | --- |
| 1 | Selection bias – was a sufficiently random sequence generated to produce comparable groups? |
| 2 | Selection bias – was allocation concealment performed to sufficient standards? |
| 3 | Performance bias – was the blinding of participants performed to sufficient standards? |
| 4 | Detection bias – was the blinding of outcome assessment performed to sufficient standards? |
| 5 | Attrition bias – how complete was the handling of outcome data for each main outcome, including reporting and explanations for attrition or exclusions? |
| 6 | Reporting bias – were there any instances reporting bias derived from selective outcome reporting? |
| 7 | Any other biases observed? |

The risk of bias assessed in response to each question was scored either as a ‘high’, ‘low’, or an ‘unclear’.

|  |  | RCT Referenced (listed by First Author) | | | | | | | | | |
| --- | --- | --- | --- | --- | --- | --- | --- | --- | --- | --- | --- |
|  |  | Koga 1988 | Hamazoe 1994 | Fujimura 1994 | Fujimoto 1999 | Yang 2011 | Spiliotis 2015 | Driel 2018 | Klaver 2019 | Goere 2020 | Quenet 2021 |
| Assessment Question | 1 | L | L | U | L | L | L | L | L | L | L |
|  | 2 | L | L | L | L | L | U | L | L | L | L |
|  | 3 | U | H | U | U | U | U | U | H | H | H |
|  | 4 | L | L | L | L | L | L | L | L | L | L |
|  | 5 | L | L | L | L | L | L | L | L | L | L |
|  | 6 | L | L | L | L | L | L | L | L | L | L |
|  | 7 | L | L | L | L | L | H | L | U | L | L |
| Total L count | | 6 | 6 | 5 | 6 | 6 | 4 | 6 | 5 | 6 | 6 |

**Supplementary Table S2** – Summary of quality assessment rubrics given by the Methodological Index for Non-Randomised Studies (MINORS) for non-RCTs

| No. | Assessment Criteria |
| --- | --- |
| 1 | A clearly stated aim |
| 2 | Inclusion of consecutive patients |
| 3 | Prospective collection of data |
| 4 | Endpoints appropriate to the aim of the study |
| 5 | Unbiased assessment of study endpoints |
| 6 | Follow-up period appropriate to the aim of the study |
| 7 | Less than 5% of cohort loss to follow up |
| 8 | Prospective calculation of the study size |
| 9 | Presence of an adequate control group |
| 10 | Control and studied group managed during the same time period |
| 11 | Baseline equivalence/homogeneity of groups |
| 12 | Adequate and relevant statistical analyses |

Each question was assessed with a 3-point scale from 0 to 2. The grading scheme is as follows: 0 – not reported; 1 – reported but inadequate; 2 – reported and adequate.

|  |  | Research Paper Referenced (listed by First Author) | | |
| --- | --- | --- | --- | --- |
|  |  | Hirose 1999 | Bonnot 2019 | Ceresoli 2018 |
| Assessment Question | 1 | 2 | 2 | 2 |
|  | 2 | 2 | 2 | 2 |
|  | 3 | 2 | 2 | 2 |
|  | 4 | 2 | 2 | 2 |
|  | 5 | 0 | 1 | 1 |
|  | 6 | 2 | 2 | 2 |
|  | 7 | 2 | 2 | 2 |
|  | 8 | 2 | 2 | 2 |
|  | 9 | 2 | 2 | 1 |
|  | 10 | 2 | 1 | 1 |
|  | 11 | 2 | 1 | 1 |
|  | 12 | 2 | 2 | 2 |
| Points | | 22 | 21 | 20 |

**Supplementary Table S3** – Summary of assessment of certainty/confidence rubrics given by the GRADE guidelines

| No. | Assessment Criteria |
| --- | --- |
| 1 | Study limitations |
| 2 | Inconsistency of results |
| 3 | Indirectness of evidence |
| 4 | Imprecision of evidence |
| 5 | Presence of reporting bias |

Each question was assessed subjectively, eventually summarising the available certainty of data with help of a 4-point scale from 1 to 4. The grading scheme is as follows, as suggested by the GRADE working group:

4 – high quality, further research is very unlikely to change our confidence in the estimate of effect;

3 – moderate quality, further research is likely to have an important impact on our confidence in the estimate of effect and may change the estimate;

2 – low quality; further research is very likely to have an important impact on our confidence in the estimate of effect and is likely to change the estimate

1 – very low quality, any estimate of effect is very uncertain

**Studies on gastric cancer**

| **No. of studies** | **Study design** | **Study limitations** | **Inconsistency of results** | **Indirectness of evidence** | **Imprecision of evidence** | **Reporting bias** |
| --- | --- | --- | --- | --- | --- | --- |
| 7 | RCT, nRCT, PSM cohort | Majority of studies are old and utilise outdated methodology. Studies do not cover patient disease characteristics such as CC/PCI status. Majority of studies originate from East Asia (Japan) | No significant inconsistency | No significant indirectness | Several studies fail to report significance | Unable to accurately assess, but influx of similar Japanese studies within the particular time period (90s) leave room for suspicion |
| **Overall assessment** | | ⊕⊕○○ **Low quality** | | | | |

**Studies on ovarian cancer**

| **No. of studies** | **Study design** | **Study limitations** | **Inconsistency of results** | **Indirectness of evidence** | **Imprecision of evidence** | **Reporting bias** |
| --- | --- | --- | --- | --- | --- | --- |
| 3 | RCT, PSM cohort | Spiliotis et al. did not consider DFS and peritoneal recurrence endpoints | No significant inconsistency | Spiliotis et al. utilises CRS + systemic chemo + HIPEC vs CRS + systemic chemo while the other trails utilised CRS + HIPEC vs CRSa. OVHIPEC trial dealt with patients with primary OC with synchronous PM/advanced disease while Spiliotis et al. focused on metachronous PM findings. | No significant imprecision | Spiliotis et al. has documented methodological limitations |
| **Overall assessment** | | ⊕⊕⊕○ **Moderate quality** | | | | |

**Studies on colorectal cancer**

| **No. of studies** | **Study design** | **Study limitations** | **Inconsistency of results** | **Indirectness of evidence** | **Imprecision of evidence** | **Reporting bias** |
| --- | --- | --- | --- | --- | --- | --- |
| 3 | RCT | No serious limitations | 60-day increased risk reported by PRODIGE 7 is a novel point and not reported by any other source | PROPHYLOCHIP trial utilised a second-look surgery before HIPEC, with varying HIPEC regimens. PROPHYLOCHIP and COLOPEC trial focused more on prevention of PM whereas PRODIGE 7 focused on treatment of PM. | No significant imprecision | No serious risk of bias |
| **Overall assessment** | | **⊕⊕⊕⊕ High quality** | | | | |
